# A randomized controlled trial of online symptom searching to inform patient generated differential diagnoses

**DOI:** 10.1038/s41746-019-0183-0

**Published:** 2019-11-11

**Authors:** Seth S. Martin, Emmanuel Quaye, Sarah Schultz, Oluwaseun E. Fashanu, Jane Wang, Mustapha O. Saheed, Prem Ramaswami, Hermes de Freitas, Berthier Ribeiro-Neto, Kapil Parakh

**Affiliations:** 1 Johns Hopkins University School of Medicine, Baltimore, MD USA; 2 Johns Hopkins University Bloomberg School of Public Health, Baltimore, MD USA; 3 Google, Mountain View, CA USA

**Keywords:** Diagnosis, Patient education, Health care, Signs and symptoms, Medical research

## Abstract

Patient online health searching is now commonplace, however, the accuracy of patient generated differentials for new symptoms and potential for patient anxiety are concerns. We aimed primarily to determine the accuracy of patient generated differentials for new symptoms with and without online searching, and secondarily, to evaluate the impact of searching on anxiety levels. In the waiting room prior to seeing a clinician, 300 patients with new symptoms were randomly assigned 1:1:1 to Google searching with health related features including a symptom search tool vs Google searching with health related features disabled vs no searching. Participants were 18 years or older and presenting to the emergency department of an urban academic medical center with new low-acuity symptoms that were not due to exacerbation of a chronic condition. Search groups received access on a tablet/smartphone to Google searching with or without health related features. Both search groups could access any websites; health related features led the patient to common diagnoses and physician-validated information. The primary outcome was accuracy of the patient generated differential assessed by matching at least two of the top three diagnoses on the clinician’s differential. A secondary outcome was anxiety by a visual analogue scale. Patients were a median of 33.1 (IQI 26.2–45.9) years old, 60% women, 63% black, 82% had a high school education or less, and 45.7% reported having performed an online search prior to presentation. Search group patients spent a median of 3.82 (2.53–5.72) minutes searching online. Similar proportions of patients in each group matched at least two of three clinician diagnoses: 27.0% and 28.3% for Google searching with and without health related features vs 23.8% in the no search group. Patients in the search groups had a similar odds of matching ≥2/3 diagnoses as the no search group [OR (95% CI): 1.23 (0.70–2.13), *p* = 0.47]. Anxiety was unchanged with online searching. In conclusion, brief online searching in the waiting room did not improve accuracy of patient generated differential diagnoses for new symptoms. The absence of an increase in patient anxiety provides reassurance for subsequent work to refine and investigate online symptom search tools.

## Introduction

The diagnostic process is central to medicine and has traditionally been regarded as a responsibility of the physician. Though the medical community typically regards the processes of differential diagnosis formation and evaluation to begin in the clinical space, patients may begin to conceive of possible explanations as soon as symptoms arise, before seeing a clinician. In the digital age, wherein patients possess immediate and largely unrestricted access to a broad spectrum of online information to supplement pre-existing knowledge of relevant health conditions and to explain new symptoms, patient online health searching is commonplace. An estimated 72% of Internet users in the United States have searched online for health information, mostly through search engines, such as Google, Bing, and Yahoo.^1^

The ability to search for symptoms online holds the potential to bridge inherent information asymmetry between patients and clinicians. Whether searching occurs when one is at home, in the waiting room at a hospital, or elsewhere, patients who bring a working list of potential differential diagnoses produced from an online search into their clinical encounter could theoretically collaborate in the diagnostic process more efficiently and effectively. This, is turn, could accelerate their diagnostic workup and initiation of effective clinical care.

There have been no randomized clinical trials demonstrating the effects of online searching and health care outcomes. Previous observational/cross-sectional studies suggest that online searching could increase patient engagement, promote patient-centered care, improve patient understanding, and enable patient discussions with clinicians.^2–5^ However, concerns have been raised that online symptom checkers can give inaccurate advice,^6^ and that they could induce patient anxiety.^3,7,8^ Therefore, clinical trial evidence is needed to resolve uncertainty in the effects of online searching.

Google is the leading internet search engine and has iterated on its search experience by adding health related features including a mobile symptom search tool. We conducted a randomized controlled trial to investigate if online searching in newly symptomatic patients can increase the accuracy of patient differential diagnosis generation. We further evaluated changes in anxiety and the patient–clinician relationship.

## Results

### Patient characteristics

We assessed 546 patients for eligibility and enrolled 300 newly symptomatic patients (Supplementary Fig. 2) with no clinically meaningful imbalances between randomized groups (Table 1). Overall, patients had a median (IQI) age of 33.1 (26.2–45.9) years, 59.7% were women, 63.3% were black, 42.0% full-time employees, 82.0% had a high school education or less, and 72.0% had an annual household income of <$45,000. Nearly all participants (98.3%) owned a smartphone; 33.3% owned an Apple device while 65.0% owned an Android device. Nearly half (45.7%) of patients reported performing an online symptom search prior to arriving in the ED and 92.7% used Google as their main search engine. While in the waiting room prior to seeing the clinician, search group patients spent a median of 3.82 (2.53–5.72) minutes on online symptom searching, with no difference between search groups (*p* = 0.26). Only three participants (1.5%) spent <1 min in search time.

**Table 1:** Characteristics of trial patients

Characteristics	Overall (*n* = 300)	Google search (*n* = 100)	HFD (*n* = 99)	No search (*n* = 101)
Age, y, Median (IQI)	33.1 (26.2–45.9)	30.6 (24.6–47.1)	35.3 (28.8–46.6)	33.9 (26.2–44.8)
Sex, no. (%)				
Women	179 (59.7)	60 (60.0)	53 (53.5)	66 (65.4)
Men	121 (40.3)	40 (40.0)	46 (46.5)	35 (34.7)
Race, no. (%)				
White	89 (29.7)	33 (33.0)	28 (28.3)	28 (27.7)
Black	190 (63.3)	62 (62.0)	65 (65.7)	63 (62.4)
Other	21 (7.0)	5 (5.0)	6 (6.1)	10 (9.9)
Employment, no. (%)				
Full-time	126 (42.0)	38 (38.0)	48 (48.5)	40 (39.6)
Part-time	41 (13.7)	16 (16.0)	8 (8.1)	17 (16.8)
Retired	11 (3.7)	4 (4.0)	3 (3.0)	4 (4.0)
Unemployed	122 (40.7)	42 (42.0)	40 (40.4)	40 (39.6)
Enrollment site, no. (%)				
JHBV ED	137 (45.7)	43 (43.0)	44 (44.4)	50 (49.5)
JHH ED	163 (54.3)	57 (57.0)	55 (55.6)	51 (50.5)
Educational level, no. (%)				
Some high school or less	64 (21.3)	20 (20)	21 (21.2)	23 (22.8)
High school or GED	182 (60.7)	62 (62)	58 (58.6)	62 (61.4)
College or university degree	38 (12.7)	16 (16)	11 (11.1)	11 (10.9)
Master’s degree	8 (2.7)	1 (1)	5 (5.1)	2 (2)
Doctorate	8 (2.7)	1 (1)	4 (4)	3 (3)
Annual household income ($), no. (%)				
<45,000	216 (72.0)	72 (72.0)	69 (69.7)	75 (74.3)
45,000 to <60,000	45 (15.0)	17 (17.0)	17 (17.2)	11 (10.9)
60,000 to <100,000	20 (6.7)	5 (5.0)	9 (9.1)	6 (5.9)
≥100,000	19 (6.3)	19 (6.3)	4 (4.0)	9 (8.9)
Type of work (US Census categories)				
Management/professional	45 (15.0)	12 (12.0)	17 (17.2)	16 (15.8)
Service	64 (21.3)	24 (24.0)	15 (15.2)	25 (24.8)
Sales/office	25 (8.3)	10 (10.0)	10 (10.1)	5 (5.0)
Construction/maintenance	26 (8.7)	6 (6.0)	11 (11.1)	9 (8.9)
Other	24 (8.0)	7 (7.0)	8 (8.1)	9 (8.9)
Smartphone				
iPhone owner, no. (%)	100 (33.3)	34 (34.0)	30 (30.3)	36 (35.6)
Android owner, no. (%)	195 (65.0)	64 (64.0)	65 (65.7)	66 (65.4)
Online symptom searching prior to visit, no. (%)				
Searched symptoms online prior to ED	137 (45.7)	46 (46.0)	41 (41.4)	50 (49.5)
Used Google as main search engine	278 (92.7)	93 (93.0)	92 (92.9)	93 (92.1)
Search time in ED, Median (IQI), minutes	3.82 (2.53–5.72)	4.13 (2.64–6.03)	3.45 (2.48–5.10)	–

*IQI* inter-quartile interval, *JHBV ED* Johns Hopkins Bayview Emergency Department, *JHH ED* Johns Hopkins Hospital Emergency Department, *HFD* Health related features disabled

### Clinician characteristics

Thirty-one clinicians participated in this trial, including 58.1% women, 80.0% whites, with a median age of 46.5 (35.7–50.3) years and most clinicians (74.2%) having at least 6 years of experience post-training. Regarding types of clinicians, 41.9% were physicians, 6.5% were nurse practitioners, and 51.6% were physician assistants. All clinicians owned a smartphone; 90.3% owned an Apple device while 9.7% owned an Android device.

Clinicians commonly sought medical information from digital sources such as UpToDate (100%) and internet searching (80.7%) (Table 2). However, clinicians tended to disagree when asked on a scale of 0 (disagree) to 100 (agree) if they liked when patients brought results from internet searches to visits [median response 16.5 (IQI: 1–50)] and if they recommend specific websites to patients [median response 21 (IQI: 1–50)].

**Table 2:** Characteristics of trial clinicians

Characteristics	Clinicians (*N* = 31)
Age, y, Median (IQI)	46.5 (35.7–50.3)
Sex, no. (%)	
Women	18 (58.1)
Men	13 (41.9)
Race, no. (%)	
White	24 (80.0)
Black	2 (6.7)
Other	4 (13.3)
Professional degree, no. (%)	
MD	13 (41.9)
NP	2 (6.5)
PA	16 (51.6)
Board certification, among MDs	
Internal Medicine	1 (7.7)
Family Medicine	1 (7.7)
Emergency Medicine	8 (61.5)
Others	3 (23.1)
Years out of training, no. (%)	
<2 years	5 (16.1)
2–5 years	3 (9.7)
6–10 years	6 (19.4)
11–20 years	12 (38.7)
>20 years	5 (16.1)
Primary site of clinical work, no. (%)	
GIM	1 (3.2)
JHBV ED	21 (67.8)
JHH ED	9 (29.0)
Hours per shift, Median (IQI)	12 (9–12)
Patients per shift, Median (IQI)	18 (15–22)
Number of shifts per week, Median (IQI)	2 (1–3)
Clinician likes when patients bring results from internet search (disagree to agree; 0–100), Median (IQI)	16.5 (1–50)
Clinician recommends specific websites for patients (disagree to agree; 0–100), Median (IQI)	21 (1–50)
Clinician recommends smartphone apps to patients (disagree to agree; 0–100), Median (IQI)	2 (0–7)
Owns a smartphone or tablet, no. (%)	31 (100)
iPhone or iPad, no. (%)	28 (90.3)
Android device, no. (%)	3 (9.7)
Sources of medical information, no. (%)	
Textbooks	18 (58.1)
Journals	21 (67.7)
Internet search	25 (80.7)
UpToDate	31 (100)
Other online resource such as Epocrates	15 (48.4)
Medical app on phone such as Micromedex	15 (48.4)

*GIM* Johns Hopkins Green Spring Station General Internal Medicine, *JHBV* Johns Hopkins Bayview ED, *JHH ED* Johns Hopkins Hospital Emergency Department, *MD* medical degree, *NP* nurse practitioner, *PA* physician assistant, *SD* standard deviation

### Distribution of diagnoses

The most common primary diagnosis (*n* = 300) made by the clinicians was musculoskeletal in nature (32.7%). This was followed by upper respiratory tract infections (12.0%), dental diseases (8.3%), diseases of the skin and soft tissue (8.3%), and gastrointestinal diseases (7.3%). All clinicians provided a primary diagnosis while 99.3% (*n* = 298) of patients did. The proportion of second and third differential diagnoses left unfilled were 9% and 30%, respectively, for clinicians and 9% and 30%, respectively, for patients.

### Diagnoses match

Similar proportions of patients in each trial group matched at least 2/3 diagnoses: 27.0% vs 28.3% vs 23.8% for the Google Search vs HFD vs no search group (Table 3a). The odds of matching at least 2/3 diagnoses was not significantly different between patients in the two search groups vs the no search group [odds ratio (95% CI): 1.23 (0.70–2.13), *p* = 0.47]. Furthermore, there was no evidence that searching versus not searching prior to presenting modified our primary outcome (p for interaction 0.63). There was also no difference in the odds of matching at least 1/3 diagnosis [1.27 (0.74–2.17)] and all three diagnoses [2.58 (0.30–22.36)] (Table 3b). The odds of having 2/3 matches in participants in the Google Search group was not significantly different than in the HFD group [0.94 (0.50–1.75)]. Respective comparisons for matching one or all three diagnoses were 1.58 (0.82–3.04) and 1.50 (0.25–9.18) (Table 3b).

**Table 3:** Accuracy of patient generated pre-visit differential diagnosis compared with clinician differential, by (A) Proportion of matched diagnoses and (B) Odds ratios (95% CI) for matched diagnoses between groups

A					
Diagnostic accuracy (%)	Overall (*n* = 300)	Google search (*n* = 100)	HFD (*n* = 99)	No search (*n* = 101)	*p*-value
Primary outcome (≥2/3 matched)	79 (26.3)	27 (27.0)	28 (28.3)	24 (23.8)	0.76
Sensitivity analysis					
3/3 matched	6 (2.0)	3 (3.0)	2 (2.0)	1 (1.0)	0.62
≥1 matched	223 (74.3)	80 (80.0)	71 (71.7)	72 (71.3)	0.28

B				
Diagnostic accuracy (Odds ratio (95% CI)	Search group^a^ (*n* = 199)	*p*-value	Google search^b^ (*n* = 100)	*p*-value
Primary outcome (≥2/3 matched)	1.23 (0.70–2.13)	0.47	0.94 (0.50–1.75)	0.84
Sensitivity analysis				
3/3 matched	2.58 (0.30–22.36)	0.39	1.50 (0.25–9.18)	0.66
≥1 matched	1.27 (0.74–2.17)	0.39	1.58 (0.82–3.04)	0.17

Search group = Google + HFD

^a^compared to No Search group (ref. ^1^)

^b^compared to HFD group (ref. ^1^)

### Baseline and change in anxiety scores

The median (IQI) anxiety scores [calm (0) to anxious (30)] at baseline for the Google Search, HFD, and No Search groups were 25 (15–30), 20 (12–30), and 25 (13–30), respectively. The post-visit anxiety scores of the Google Search [15 (1–30)] and No Search [15 (2–27)] groups were significantly lower than their baseline scores (both *p* < 0.001) while the HFD group [20 (10–30)] did not show a significant change in anxiety scores (*p* = 0.18) (Table 4).

**Table 4:** Change in anxiety score post-visit vs pre-visit

Group	*n*	Median (IQI) pre	Median (IQI) post	Median Change (IQI)	*p*-value^a^
Google Search	100	25 (13–30)	15 (1–30)	0 (−12.5 to 0)	0.0001
HFD	99	20 (12–30)	20 (10–30)	0 (−7 to 0)	0.184
No Search	101	25 (15–30)	15 (2–27)	0 (−15 to 0)	<0.0001

^a^*P*-values derived from Wilcoxon signed rank sum test

### Patient–clinician relationship

There were no differences between randomized groups in overall satisfaction with care, communication, feeling connected, shared decision making, or other aspects of the patient–clinician relationship, as reported by either patients or clinicians.

At the conclusion of the office visit [worse than usual (0); usual (50); better than usual (100)], participants felt satisfied with their care [median (IQI): 75 (50–100)], the speed of diagnosis [80 (50–100)], length of visit [61 (50–100)], and shared decision-making [75 (50–100)]. They tended to have a better than usual communication [80 (50–100)] and feeling of connection [75 (50–100)] with their clinicians (Supplementary Table 1).

Clinicians tended to be more conservative in their responses to similar questions assessing their relationship with the patients. They reported usual satisfaction with patient care provided [51 (50–75)], length of visit [50 (50–61.5)], and shared decision-making [51 (50–79.5)]. They also reported a better than usual communication with patients [60 (50–80)], connection with patients [55 (50–81)], and speed of diagnosis [57 (50–80)]. Only 13 (4.3%) patients brought up information they found online during their visit with 69.2% of them being concerned about diagnoses that were in the clinician’s differential. The clinicians also felt that the information provided by these 13 patients made a positive impact on shared decision-making (Supplementary Table 1).

## Discussion

This randomized controlled trial evaluated the effect of patient online searching prior to engaging with a clinician. Contrary to our hypothesis, online searching in the emergency department did not result in patients being more likely to identify at least two items on their differential diagnosis that matched with the clinician’s differential.

We found that over 70% of patients, regardless of whether they were randomized to searching online or not, identified one diagnosis on their differential that matched their clinician’s differential. However, far fewer patients identified two or three matching items on their differential, with similar results by search group. We suspect that the lack of effect may be related, in part, to a limited set of queries triggering health features in the study environment. Patients needed to enter an exact symptom, disease script, or diagnosis, manually or using autocomplete, to trigger knowledge panels. For example, a search for “chest pain” triggered health related features whereas a search of “my chest has been hurting for 2 weeks” did not. It has since been updated on Google Search, and now does.

The diminished differences among the groups could also be because almost half of patients in all groups had already investigated their symptoms online, mainly on Google, before coming to the ED. Alternatively, the null results could be related to education level; 82% of patients had a high school education or less. Furthermore, the lack of effect in this trial may relate to differential diagnosis completeness. Although clinicians and patients were instructed to list the top three most likely diagnoses, 9% in each group left the second possible diagnosis place unfilled and 30% left the third unfilled. As such, they had less opportunity to have a match. Nevertheless, given that generating a differential diagnosis is a medical framework, it is encouraging that patient generated lists were as complete as those of clinicians.

Considering the depth of searching, Google’s health search environment provides functionality to click on a health condition to learn more than surface level information. We observed that no patients clicked on conditions to learn more. In the context of an acute condition, patients may be distracted by their health concern and desire immediate medical attention rather than first understanding the condition themselves. A patient with more chronic symptoms at home or in an ambulatory clinic waiting room, on the other hand, might spend more time conducting an online search and might explore more health related features of Google Search. Therefore, it is important to consider that our emergency department based search study does not address effects of searching for the many individuals who search daily in other contexts.

Clinicians in this trial were on the younger side and all owned personal smartphone devices, mostly Apple iPhones. They commonly searched themselves for medical information on the internet and UpToDate. However, clinicians tended to hold negative views toward patients bringing results from internet searching and generally did not recommend specific websites or smartphone applications to patients. These views could limit clinician acceptance of patient directed online health searching and warrant further study.

Still, the approach of introducing searching in the waiting room was successfully implemented within routine clinical workflow. Moreover, there was no appreciable worsening of the patient–clinician relationship and the absence of an increase in anxiety is reassuring given concerns raised in prior literature.^9–11^ Further work on online symptom searching is needed as our trial tested an early iteration of one tool in a specific setting at a single institution.

The limitations of this trial should be considered. Matching between patient and clinician differential diagnoses was determined by physician review, which may be imprecise and introduce bias. On the other hand, no objective, validated matching algorithm exists, and physician review for matching was done with blinding to group assignment and independently by three separate physicians. This trial may lack generalizability as it tested one online searching platform at a specific time in one clinical setting in a single health system. Future randomized clinical trials in this field of study are warranted to investigate other searching platforms at different time points in the workflow of patient care in other settings. Additionally, we captured online searching on trial-issued devices to standardize the experience and could not account for any searching that may have occurred on personal devices; future studies may consider obtaining consent to capture searching on personal devices. Given that our patient and clinician satisfaction measures were not previously validated, these measures should be interpreted with caution. Finally, nearly half of participants had searched online prior to presenting to the ED, which may reduce any potential impact of introducing searching in the waiting room.

In conclusion, in newly symptomatic patients presenting to the emergency department, online searching before seeing a clinician did not yield a benefit in the accuracy of the patient generated differential diagnosis. However, the absence of an increase in patient anxiety provides reassurance in continued efforts to bridge information asymmetry between patients and clinicians through online delivery of health information.

## Methods

### Trial design, participants, and eligibility

This was a parallel group trial randomizing patients in a 1:1:1 ratio to Google Search with or without health related features vs No Search. Ethics committee approval was obtained from the Johns Hopkins IRB and written informed consent was obtained from participants. Patients, along with their clinician, were recruited from the Johns Hopkins Hospital and Johns Hopkins Bayview Medical Center Emergency Departments in Baltimore, Maryland. Study enrollment began 7 September 2016 and final data collection ended 15 August 2017. We conducted an observation phase to examine Emergency Department waiting room activities and then a run-in phase to evaluate feasibility/usability in 30 patients, refine our study design, and improve clarity of surveys. Informed by these preliminary phases, on 8 March 2017 we registered (ClinicalTrials.gov number, NCT03073746) and launched the main trial of 300 patients as reported here. Data were collected in Microsoft Excel Version 16.

We included patients aged 18 years or older presenting to the emergency department with a new unexplained symptom or group of symptoms and triaged to ESI level 3–5 (low acuity). We excluded patients presenting with an exacerbation of a chronic condition defined by the perception of the patient. That is, if a patient for example had respiratory symptoms felt by the patient to be an asthma exacerbation, such a patient did not qualify. We also excluded those who were not literate (defined as self-reported inability to read and write), non-English Speaking, not mentally competent to provide consent due to inability to understand relevant information due to deficit in intelligence (e.g., developmental disability), memory (e.g., advanced dementia or significant delirium), or attention span (e.g., Attention Deficit Disorder (ADD) or mania) based on prior documentation in medical records or as judged by the researchers, or unable to use a phone/tablet for any mental or physical impairment (e.g., blind).

### Interventions

The first search group received access on a tablet/smartphone to Google searching with health related features including a symptom search tool that leads users to knowledge cards (Google Search). These knowledge cards deliver medical information such as typical symptoms, diagnoses, treatments, and medical illustrations. The information has been compiled, curated, and reviewed for accuracy by physicians from leading institutions and is ranked by the probability of a health condition. Screenshot examples of the mobile Google search with health related features are provided in Supplementary Fig. 1. The second search group received access on a tablet/smartphone to Google Search with health related features disabled (HFD). The search groups were instructed to perform the search to inform what diagnoses they thought were most likely causing their symptoms and to consider questions that they may want to ask their clinician. Each search group was allotted a maximum of 15 min for search time before seeing the clinician. However, the investigators anticipated that the typical patient would search for <5 min and judged that as a meaningful amount of time to review information and form a differential.

### Outcomes

The primary outcome was accuracy of the patient generated differential diagnosis assessed by matching ≥2/3 diagnoses with the clinician differential. The clinician differential was generated immediately post history and physical examination. The denominator was fixed at 3 as the patient and the clinician each could list a maximum of three diagnoses. To determine the numerator, three physician reviewers (OF, EM, SM) independently evaluated differential diagnoses and scored them as 0, 1, 2, or 3 based on the number of unordered matches made between the patient and clinician diagnoses. Therefore, 2/3 agreement meant that the patient and clinician listed 2 of 3 diagnoses that were the same, regardless of where they were on each list. Disagreements (2%) were resolved through discussion to reach consensus.

A pre-specified secondary outcome was anxiety, scored from 0 (calm) to 30 (anxious) per the visual analogue scale (VAS-A), which is validated for quick repetition.^12^ An exploratory secondary outcome was the patient–clinician relationship, assessed by post-visit surveys of patients and clinicians. The surveys assessed on a 0 (worse than usual) to 100 (better than usual) scale overall satisfaction, communication, feeling connected, speed of diagnosis, visit length, and shared decision making. These surveys have not been previously validated or historically collected at our recruitment sites. No changes were made to outcomes after trial commencement.

### Sample size

We hypothesized that patients in the search groups would have a higher percentage of matching 2/3 diagnoses made by the clinician compared to patients who did not search and estimated that 300 patients would give 80% power to detect an absolute difference of 20% or more. There were no early stopping rules.

The VAS-A is reported to have a mean of 8.6 and standard deviation of 7.4. With 100 patients in each group, there was 80% power to detect a difference of 3.0 or higher.

### Randomization and blinding

Patients were randomized using a block size of 20, as determined by a random number generator by EQ, with concealment of randomization sequence until intervention assignment. EQ and SS enrolled patients and assigned them to interventions. Blinding of patients was not possible but patients were asked not to reveal their group assignment to their clinician. Investigators were blinded to assignments when assessing outcomes.

### Statistical analyses

No data were excluded from the analyses. We present continuous variables as medians (interquartile interval: IQI) and categorical variables as frequency (percentage). We made comparisons across the intervention groups using appropriate statistical tests; Kruskal-Wallis tests for medians, and *χ*^2^ test or Fisher’s exact test for discrete variables. In our primary analysis, using an intention-to-treat approach, we used logistic regression models to determine the odds of having 2/3 matches between search and no search groups. We performed an interaction analysis comparing participants who reported searching versus not searching prior to presenting to the hospital. As a sensitivity analysis, we also explored the odds of having 1/3 matches and 3/3 matches between groups. We used Wilcoxon signed rank sum test to assess changes in baseline anxiety scores. We considered *p*-values < 0.05 to be statistically significant and performed our analyses using Stata Version 14.

### Reporting summary

Further information on research design is available in the Nature Research Reporting Summary linked to this article.

## Supplementary information

Supplementary Information

Reporting Summary Checklist

## Data Availability

The datasets generated during and/or analyzed during the current study are available from the corresponding author on reasonable request.
