# Supplementary material for: A randomized controlled trial of online symptom searching to inform patient generated differential diagnoses
Source: NPJ Digit Med. 2019 Nov 11;2:110. doi: 10.1038/s41746-019-0183-0 (PMC6848180; doi:10.1038/s41746-019-0183-0)
Supplement: Supplementary file 1 — Supplementary Information [file 41746_2019_183_MOESM1_ESM.pdf]

Supplement

Table of contents

Supplementary Figure 1 – page 2

Supplementary Figure 2 – page 3

Supplementary Table 1 – page 4-5

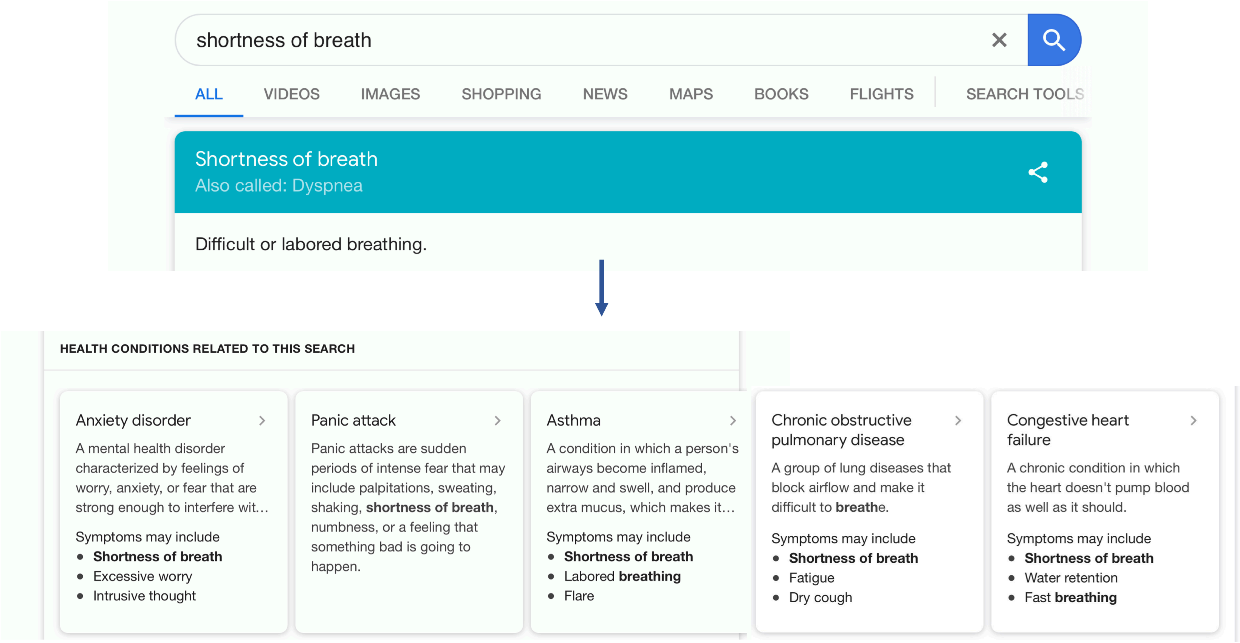

Supplementary Figure 1. Screenshot example of mobile Google search with health related features. When one searches on a mobile device for a symptom, such as “shortness of breath” or “trouble breathing”, one can scroll through health conditions related to the search (or, in other words, possible diagnoses for the symptom or symptoms). Google and the Google logo are registered trademarks of Google LLC, used with permission.

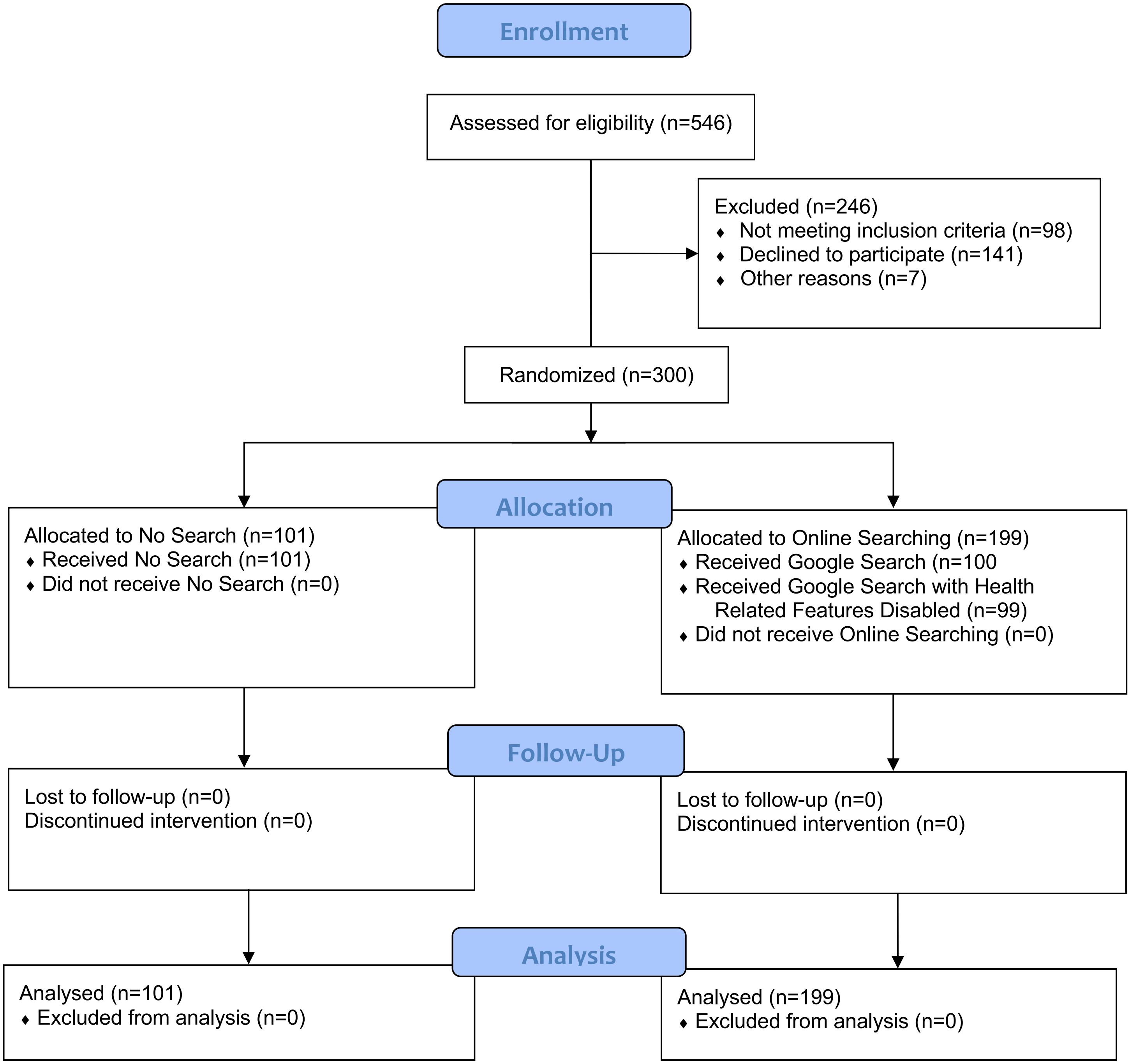

Supplementary Figure 2. CONSORT flow diagram.

Supplementary Table 1. Quality of patient-clinician relationship as assessed post-visit by elements of patient satisfaction, clinician satisfaction, and clinician perception of online searching.

	Median (interquartile interval) response score				
	Overall (n=300)	No Search (n=101)	HFD (n=99)	Google Search (n=100)	p-value
Patient satisfaction (range 0 – 100, from worse to better than usual)					
Satisfaction with the care you received	75 (50 - 100)	80 (50 - 100)	80 (50 - 100)	64.5 (50 - 100)	0.31
Communication with clinician	80 (50 - 100)	80 (50 - 100)	89 (50 - 100)	80 (50 - 100)	0.71
Feeling connected with clinician	75 (50 - 100)	75 (50 - 100)	80 (50 - 100)	75 (50 - 100)	0.92
Speed of diagnosis	80 (50 - 100)	80 (50 - 100)	80 (50 - 100)	75 (50 - 100)	0.81
Length of visit	61 (50 - 100)	70 (50 - 100)	60 (50 - 100)	53.5 (50 - 90)	0.24
Shared decision making	75 (50 - 100)	70 (50 - 100)	80 (50 - 100)	77.5 (50 - 100)	0.78
Clinician satisfaction (range; 0 – 100, from worse to better than usual)					
Satisfaction with the care you provided	51 (50 - 75)	52 (50 - 80)	50 (50 - 74)	51 (50 - 70)	0.46
Communication with patient	60 (50 - 80)	60 (50 - 85)	53.5 (50 - 80)	60 (50 - 80)	0.45
Feeling connected with patient	55 (50 - 81)	60 (50 - 85)	51 (50 - 80)	59 (50 - 80.5)	0.69
Speed of diagnosis	57 (50 - 80)	55 (50 - 80)	56 (50 - 70)	60 (50 - 80)	0.56
Length of visit	50 (50 - 61.5)	52 (50 - 70)	52 (50 - 60)	50 (50 - 60)	0.27
Shared decision making	51 (50 - 79.5)	52 (50 - 80)	51 (50 - 75)	50 (50 - 71.5)	0.34
Clinician perceptions					

I felt the patient challenged my authority, n (%)	8 (2.7)	4 (4.0)	2 (2.0)	2 (2.0)	0.74
Patient brought up information during the visit that they found online	13 (4.3)	3 (3.0)	4 (4.0)	6 (6.0)	0.57
Which of the following statements best describes the information that the patient brought up?[a]					0.26
The patient was concerned about diagnoses that were in my differential	9 (69.2)	2 (66.7)	4 (100)	3 (50.0)	-
The patient was concerned about misleading/inappropriate diagnoses	0 (0)	0 (0)	0 (0)	0 (0)	-
Others	4 (30.8)	1 (33.3)	0 (0)	3 (50.0)	-
What impact did that information have on shared decision making? (range 0 – 100, from negative to positive)	74 (50 - 85)	74 (65 - 80)	50 (50 - 75)	82.5 (50 - 89)	0.61

[a]Of the 13 participants who brought up information during the visit that they found online
